# Evaluation of the Universal Prevention Program Klasse2000 in Fourth Grade Primary School Children: Protocol for a Propensity Score-Matching Approach

**DOI:** 10.2196/14371

**Published:** 2020-08-20

**Authors:** Sören Kliem, Yvonne Krieg, Anna Lohmann, Thomas Mößle

**Affiliations:** 1 Ernst-Abbe-Hochschule Jena Germany; 2 Criminological Research Institute of Lower Saxony Hannover Germany; 3 State Police College of Baden-Wuerttemberg Villingen-Schwenningen Germany

**Keywords:** Klasse2000, prevention program, student survey, propensity score matching, evaluation

## Abstract

**Background:**

Klasse2000 is the most widely adopted school-based prevention program in Germany. It addresses health promotion, addiction, and violence prevention in primary schools. As a universal prevention program, it has reached more than 1.4 million German children in the past 25 years.

**Objective:**

The effectiveness of Klasse2000 will be evaluated with a large representative survey among students. Students who have participated in the prevention program (intervention group) will be compared with students who did not participate (control group). The comparison will cover the following outcome domains: well-being, self-esteem, emotion regulation, food habits, behavioral problems, and school and classroom atmosphere. Furthermore, victimization and perpetration regarding bullying, alcohol consumption, smoking, and media consumption are assessed.

**Methods:**

To control for potential group differences, treatment effects will be estimated using propensity score-matching, which matches students from the intervention and control groups based on an identical propensity score or a propensity score that does not differ by more than a previously defined distance. The treatment effect will then be estimated in the matched sample taking the matching process into account.

**Results:**

Enrollment of schools began in March 2017. A total of 6376 students participated in the survey (n=4005 in control group; n=2371 in Klasse2000). The parent survey was returned by 52.13% (3324/6376) of parents. Results are expected in mid-2020.

**Conclusions:**

The results on the effectiveness of the Klasse2000 prevention program will form an empirical basis for legitimizing universal prevention programs and for planning future prevention approaches.

**Trial Registration:**

German Clinical Trials Register DRKS00014332; https://tinyurl.com/y2trvq4p

**International Registered Report Identifier (IRRID):**

DERR1-10.2196/14371

## Introduction

### Health Risks of Children and Adolescents

Although most children in Germany can be regarded as physically and mentally healthy, there are numerous studies that report alarming findings regarding pediatric health risks. The study of the health of children and adolescents in Germany (KiGGS), for example, reports that 15.0% of all primary school children are overweight. Among these, 6.4% require treatment due to obesity [1]. According to a Bundeszentrale für Gesundheitliche Aufklärung (BZgA) study, 10.6% of adolescents aged 12 to 17 years consume alcohol at least once per week, and 14.1% drink excessively at least once per month (equaling four glasses of liquor per occasion in girls and five glasses in boys) [2]. Furthermore, 9.7% of adolescents aged 12 to 17 years smoke [2]. Over 20% of children and adolescents aged 7 to 17 years suffer from mental health issues, the most prevalent being behavioral problems, anxiety, and depression [1]. An increase in mental health issues in children and adolescents has been associated with a decreased health-related quality of life [3]. Children with behavioral problems such as noteworthy results on the Strength and Difficulties Questionnaire (SDQ) [4,5] show a lower quality of life compared with children who score within the normal range [3].

Another important health-related aspect is bullying and school-based violence. A German representative survey among students from the years 2007 and 2008 showed that a significant number of 15-year-olds had been violent offenders in the previous school year. For example, 24.2% of students had hit or kicked fellow students, 34.3% had purposefully ignored others, and 51% had more than once picked on another student or said mean things [6]. Another study on violence among 9th graders in the German federal state of Lower Saxony revealed that in 2015, 17.2% of students had suffered from physical violence in the previous school year, 45.0% had been the victim of bullying, and 11.4% had encountered vandalism [7]. In a survey among 4th graders in Berlin, 33.0% of boys and 13.0% of girls showed aggressive behavior, indicating they had either hurt or threatened fellow students, committed vandalism, or played with fire [8].

According to the life skills approach [9], problematic behaviors in different domains are related and have a common cause. Life skills can be defined as skills that enable appropriate behavior in interactions with others and the ability to handle problems and stressful situations [10]. Prevention measures that are based on the life skills approach thus focus on health promotion and on strengthening the children’s social and personal resources in order to prevent addiction and violent behavior.

### Klasse2000 Program

The primary goal of the Klasse2000 program is the promotion of a healthy lifestyle in parents, children, and teachers as well as other youth workers involved in leisure activities [11]. Klasse2000 has reached over 1.4 million children in the past decades and can therefore be regarded as the most widely adapted school-based prevention program for the promotion of health and the prevention of addiction and violent behavior in German primary schools [11]. In Lower Saxony (a state in northwestern Germany with a population of about 8 million), about 2800 primary school classes with 60,000 students participated in the school year 2015-2016. This corresponds to 19.4% of the Lower Saxony student population [11,12]. The program was developed in 1991 at the Klinikum Nuremberg by an interdisciplinary workgroup around Dr Pál Bölcskei [11]. The program is sponsored by a nonprofit organization funded by donations [11].

### Domains Addressed in the Klasse2000 Intervention

For the promotion of general health and life skills, Klasse2000 addresses the following domains:

- Healthy food and beverage choices
- Exercise and relaxation
- Positive self-image and friendships
- Solving problems and conflicts
- Critical thinking and saying no (especially to alcohol and tobacco)

Specially trained Klasse2000 health promoters (with backgrounds in health care and/or education) visit the classrooms and introduce new focus areas to the lesson plan (1st grade: 2 visits; 2nd to 4th grade: 3 visits per school year). After these visits, the teacher further discusses these topics and includes them in the regular curriculum (10 to 12 units per school year) using detailed aids such as lesson guides, student worksheets, posters, CDs, and parent information material, etc.

**Figure 1:**
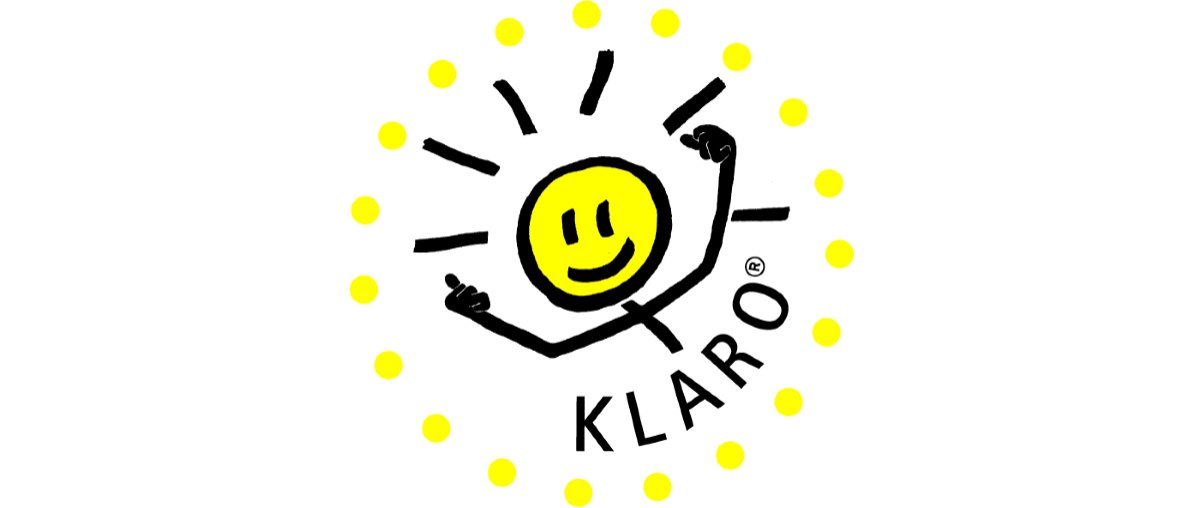
Mascot KLARO.

Furthermore, Klasse2000 includes various interactive components such as games, visits by the health promoter, visits from the mascot, KLARO (Figure 1), and special material such as a breathing coach, stethoscopes, and feeling diaries. Klasse2000 targets all students hence no special registration is required. Participation is free for schools, children, and their parents. This approach ensures that children from families at risk who have a particular high need for prevention (such as children from families with a low socioeconomic or migration background) are equally reached [12].

The parents are included using a parent newspaper, information meetings, and a yearly information letter (available in several languages). Of special importance are research tasks for the children that are accompanied by take-home material. These reinforce the topics addressed at school in the home environment. To account for individual differences, especially regarding special needs children or classes spanning varying grade levels, all student material is available for various levels of ability. The aim is to provide each child with appropriate worksheets that are neither too boring nor beyond the student’s capabilities [12]. The contents of the program are explained using the symbol figure, KLARO (Figure 1). The program developers outline the chain of the effects of the program as shown in Multimedia Appendix 1 and mention the following intended intervention effects:

- Children know their body and know what they can do to promote health and well-being, for example, regarding nutrition, exercise, and relaxation
- Children perceive health as important and are confident that they can contribute to their own health
- Children possess important life skills such as handling emotions and stress, communicating and cooperating with others, solving conflicts and saying no (eg, to alcohol and tobacco)

### Current Research Results From the Klasse2000 Prevention Program

The Klasse2000 program has previously been evaluated by the University of Bielefeld and the Institute for Therapy and Health Research (IFT-Nord). The University of Bielefeld conducted four consecutive surveys in primary school children. In their evaluation study with a randomized waiting control group design, they concluded that the dietary behavior has worsened considerably less in the intervention group. Regarding exercise, they could merely conclude that the commute to school became more passive in control group children. Furthermore, it became apparent that baseline well-being had been very high, not allowing for the detection of any differential effects. According to the parents’ perspective, the children in the intervention group had less frequently become victims of violence [13].

A study by IFT-Nord compared Klasse2000 participants to a control group of nonparticipating students in a nonrandomized control group design. Assessment took place during primary school years as well as 16 months and 3 years after the intervention. A positive effect of the Klasse2000 program on the incidence and life-time prevalence of smoking could be observed in the first and second follow-up [14,15]. A reduction in alcohol consumption was only observed immediately after the intervention and in the first follow-up. Nevertheless, the intensity of alcohol consumption among those children with previous alcohol exposure was lower in the intervention group [16]. Furthermore, health-related knowledge and classroom atmosphere were rated higher in the intervention group [17]. During primary school years, internalizing and externalizing behavioral problems were significantly reduced in classrooms participating in the Klasse2000 intervention [17]. Students participating in the intervention could be characterized as more frequently seeking social support when dealing with stress and attempting to resolve unpleasant emotions by cognitively addressing the potential root of the problem. Intervention and control groups did not differ on other health-related behavior, health-related attitudes, and life skills in the 3-year follow-up [15].

### Aim of the Evaluation

This study protocol outlines an evaluation study of the Klasse2000 intervention. Short-term effects on all four target areas are investigated via a survey among 4th grade primary students, their parents, teachers, and school principals. The evaluation of the prevention program Klasse2000 in Lower Saxony (official study title: Evaluation des Präventionsprogrammes Klasse2000 in Niedersachsen) is funded by the BZgA.

### Role of Study Sponsors

The evaluation study can be subdivided into 6 domains: (1) training of test administrators, (2) correspondence with school principals, (3) school survey, (4) data preparation and integration, (5) data analysis, and (6) dissemination of results. The sponsoring institution Kriminologisches Forschungsinstitut Niedersachsen (KFN, Criminological Research Institute of Lower Saxony) is involved in all domains, and the State Police College of Baden-Wuerttemberg is involved in domain 1. An overview of the evaluation schedule can be found in Multimedia Appendix 2. The protocol is furthermore documented according to the Standard Protocol Items: Recommendations for Interventional Trials (SPIRIT; Multimedia Appendix 3).

## Methods

### Sample and Sampling

In this evaluation study, 4th graders were assessed on the classroom level. Since 19.4% of students in Lower Saxony participate in the Klasse2000 program, it seemed feasible to base group comparisons on a sufficiently high number of students [11]. For this evaluation, a random representative sample of 600 classes was drawn out of a total of about 4000. Based on the current implementation rate of the Klasse2000 program, it was expected that around 120 of the 600 randomly selected schools were participating in the program. To ensure a sufficient rate of participating Klasse2000 classrooms, 140 additional classrooms known to have participated in the program were randomly selected and added to the sample (oversampling). In addition to surveying the 4th grade students of participating and nonparticipating classes, parents, teachers, and school principals were assessed. Overall, we aimed for the total sample to consist of 8000 students and their parents.

### Ethical Approval and Consent to Participate

The evaluation as described in this protocol has been approved by the ethics committee of the Georg Elias Müller Institute for Psychology at the Georg August University Göttingen (Multimedia Appendix 4). Furthermore, the survey was approved by the regional school authorities (Landesschulbehörde Niedersachsen H1Rb-81402-09-2017; Multimedia Appendix 5). Participation is entirely voluntary among principals, teachers, parents, and children, and nonparticipation does not lead to any negative consequences. Children whose parents have not provided written informed consent are not surveyed. Teachers administered the written informed consent forms, did not forward them to the researchers, and destroyed them 2 weeks after the survey was conducted. Since the study design provides for complete anonymization, no signature of the participating adults could be obtained for their own participation. There was no intermediary person like a teacher who could have managed the written informed consent forms, since the adult questionnaires were sent directly to the KFN. The adults gave approval for the participation in the study by sending the completed questionnaire to the institute. If a participant demands the deletion of their answers, this can be achieved up until the point the personal code (see heading Parent survey) is deleted. From then on, the data are completely anonymous and cannot be traced to an individual.

The study was registered March 22, 2018, with the German Clinical Trials Register [DRKS00014332]. The main sponsor of the study is the KFN (primary investigator SK). Furthermore, the KFN cooperates with TM of the State Police College of Baden-Wuerttemberg.

### Selection Criteria

All primary schools located in Lower Saxony and providing general education were part of the sample population. A complete list for sampling was obtained from the national office for statistics of Lower Saxony. It was not assessed whether the schools participated in any other prevention programs apart from Klasse2000. Any other prevention measures of a general or specific nature were regarded as the standard the Klasse2000 prevention program should be contrasted with. The adherence to the Klasse2000 protocol was also not essential for eligibility as this evaluation is explicitly interested in assessing the treatment effect on an intention-to-treat basis.

### Procedure

#### Administrator Training

Test administrators underwent several hours of training by the project staff of the Klasse2000 evaluation at the KFN. The training covered general information on the study, survey contents, coordination of scheduling the individual survey appointments, and professional behavior toward teachers and students. Test administrators received detailed instructions on the survey tools to ensure a standardized procedure. All test administrators were blinded regarding intervention versus control group membership of the relevant classrooms.

#### Enrollment

The schools of the selected classes were contacted by postal mail addressed to the school principal (Multimedia Appendix 6). In this initial letter, the principals were informed about the survey’s contents, potential scheduling, and which classes had been selected for participation in the evaluation study.

#### Student Survey

The student survey was conducted in a classroom context in the presence of the teacher using standardized questionnaires. A trained test administrator guided the children through the questions with the help of a fully standardized testing manual (about 28 pages). At the beginning of the survey, the children were informed of the content and nature of the questions. They were told that their participation is entirely voluntary and anonymous and that nonparticipation would not yield any negative consequences. The majority of questions and possible answers were read aloud and explained by the test administrator. Additionally, the corresponding pages were shown on a screen using an overhead or video projector. After the survey, the questionnaires were collected by the test administrator. To ensure anonymity, student and teacher questionnaires were sealed in an envelope on site and sent to the KFN by mail. Details on the assessment tools can be found in Multimedia Appendix 7.

In accordance with the World Medical Association Declaration of Helsinki, the parents and children who took part in the study were informed about our institution, the voluntary nature, aims, methods, and financing of the study by means of a parent information letter prior to the survey being conducted (Multimedia Appendix 8). The information letter stated they had the right not to take part in the survey and that there were no disadvantages if they did not participate. The same information was provided to teachers and headmasters in the letter to the principal (Multimedia Appendix 6).

#### Parent Survey

After the student survey was conducted, the children were asked to give an envelope with the parent questionnaire and a return envelope with prepaid postage to their parents. The parents could return the completed questionnaire directly to the KFN by mail. Due to the study design that provides for complete anonymization and therefore no collection of real names, no signature of the participating adults could be obtained. Parents, teachers, and principals decided on their own participation by sending the completed questionnaire to the institute. To match the children’s and the parents’ questionnaires, the children wrote a code on their parents’ questionnaire. This code is only used for matching and does not include any identifiable information. After the matching has been performed, this code will be deleted, rendering the final data completely anonymous. Due to anonymity, parents cannot be reminded to return the questionnaires nor are any incentives offered for participation.

#### Data Handling and Monitoring

Data assessment and management is completed by the KFN data protection official. The data protection official is employed directly by the KFN and has neither monetary nor scientific involvement in the project. All project staff members were obliged to uphold data protection rules and regulations. Participants do not reveal their names at any point in the study. The students provide their month of birth, age, and gender. The parents provide the same information, which will be used for matching should matching via the matching code not be possible. Children and their parents can be matched to a school by a field code. This field code is necessary to determine whether a school participates in the Klasse2000 program without endangering the blinding of the test administrators. After successful matching of parents, children, principals, and schools, all corresponding field codes are deleted. The data are then fully anonymous (ie, the school name can no longer be inferred from the cases).

### Evaluation of Effectiveness

It is of special interest whether the underlying constructs are based on the direct efficiency hypothesis of the Klasse2000 program (primary outcome) or based on an efficiency hypothesis but one that must be regarded as less probable than the primary outcome (ie, secondary outcome). For some outcomes (such as alcohol and tobacco consumption), the expected prevalence is generally low.

The evaluation focuses on the following research questions:

- Research question 1 (well-being; primary outcome domain): How does participation in the Klasse2000 prevention program influence children’s well-being?
  Hypothesis 1.1: The prevention program Klasse2000 has a positive influence on the children’s well-being.
  Hypothesis 1.2: The prevention program Klasse2000 has a positive influence on the children’s self-esteem.
  Hypothesis 1.3: The prevention program Klasse2000 has a positive influence on the children’s emotion regulation.
  Hypothesis 1.4: The prevention program Klasse2000 reduces the severity of behavioral problems.
- Research question 2 (health-related behavior; primary outcome domain): How does participation in the Klasse2000 prevention program influence children’s health-related behavior?
  Hypothesis 2.1: The prevention program Klasse2000 increases fruit and vegetable consumption.
  Hypothesis 2.2: The prevention program Klasse2000 increases the consumption of water and unsweetened tea.
  Hypothesis 2.3: The prevention program Klasse2000 decreases the consumption of sweets, salty snacks (eg, chips, pretzels), and sweetened beverages.
  Hypothesis 2.4: The prevention program Klasse2000 increases the time children spend exercising.
- Research question 3 (school and classroom atmosphere, school-based conflicts, and violence; primary outcome): How does participation in the Klasse2000 prevention program influence the school and classroom atmosphere and school-based violence?
  Hypothesis 3.1: The prevention program Klasse2000 has a positive influence on the school and classroom atmosphere.
  Hypothesis 3.2: The prevention program Klasse2000 decreases the probability of engaging in bullying.
  Hypothesis 3.3: The prevention program Klasse2000 decreases the probability of becoming a victim of bullying.
- Research question 4 (media use; secondary outcome): How does participation in the Klasse2000 prevention program influence media use?
  Hypothesis 4.1: The prevention program Klasse2000 decreases the time spent on media.
  Hypothesis 4.2: The prevention program Klasse2000 decreases the probability of watching movies with an age rating of 16 or 18.
  Hypothesis 4.3: The prevention program Klasse2000 decreases the frequency of watching movies with an age rating of 16 or 18.
  Hypothesis 4.4: The prevention program Klasse2000 decreases the probability of playing video games with an age rating of 16 or 18.
  Hypothesis 4.5: The prevention program Klasse2000 decreases the frequency of playing video games with an age rating of 16 or 18.
- Research question 5 (alcohol and tobacco consumption; secondary outcome): How does participation in the Klasse2000 prevention program influence the children’s alcohol and tobacco consumption?
  Hypothesis 5.1: The prevention program Klasse2000 decreases the probability of drinking alcohol.
  Hypothesis 5.2: The prevention program Klasse2000 decreases the frequency of alcohol consumption.
  Hypothesis 5.3: The prevention program Klasse2000 decreases the probability of smoking cigarettes.
  Hypothesis 5.4: The prevention program Klasse2000 decreases the smoking frequency.

Details on the assessment tools can be found in Multimedia Appendix 7.

### Data Analysis

The decision to participate in the Klasse2000 program is made at the school level. Hence, schools self-select for implementing the intervention. This selection bias must be controlled for when comparing the outcomes of children participating in the Klasse2000 and those who did not. The gold standard for preventing selection bias is randomization. There are three major factors that made a randomized trial highly infeasible for this evaluation: (1) as a general intervention, the Klasse2000 targets a high number of outcomes, (2) effect sizes in universal prevention programs can be expected to be rather small, (3) the intervention is implemented during the entire primary school time (ie, over a time span of 4 years). Hence, a very high number of schools would need to be randomized to participate in an intervention over several years. An attractive alternative to an randomized controlled trial is a natural experiment. However, the Klasse2000 is implemented all over Germany, and there are no immediately comparable intervention programs. Hence, neither regional implementation nor the comparison of a comparable program lend themselves for overcoming selection bias. Given these circumstances, a propensity score-matching approach will be implemented as a feasible alternative. The basic idea of a matching approach is as follows: for each student who is currently participating in the Klasse2000 program, a control student will be selected who matches the treatment group participant as closely as possible regarding criteria that are relevant yet unrelated to the participation in the prevention program (ie, gender, socioeconomic status). The matching will be performed with a matching algorithm that assigns weights to each individual in the control group (ie, inverse probability weighting). The estimation of treatment effects will be completed using regression-based analyses that take these weights into account. Simulation studies show that the propensity score-matching approach enables an unbiased estimation of intervention effects [18].

Prior to analysis, data will be checked for outliers, inconsistencies, and possible transformation. We predict that our sample size will be large enough for our statistical tests to be robust regarding nonnormally distributed variables. Power analyses (ie, a priori) based on a type I error rate (false positive) of α=.05, statistical power of 1−β=.80, and a minimum detectable effect size of ES=0.2 resulted in a required sample size of n=310 (1-tailed) students in each group (intervention and control).

Missing data will be handled using state-of-the-art imputation techniques (multiple imputation in missing non-at-random scenarios [ie, by parametric recursive partitioning] [19,20]). Wherever earlier findings suggest differential effects (ie, regarding gender), subgroup analyses of the above-mentioned hypotheses will be performed. Furthermore, post hoc analyses of different implementation factors potentially contributing to positive treatment effects over time will be conducted.

### Dissemination

Study results will be disseminated in a final report for the funder (BZgA), who will further disseminate the findings in their own publications. In addition, findings will be submitted to suitable peer-reviewed journals.

## Results

### Trial Status Upon Initial Protocol Submission

Schools were first contacted in March 2017. Between April and June 2017, students and teachers were surveyed. Teachers and principals returned their questionnaires by mail resulting in questionnaires being returned by mail until November 2017. Data entry and cleansing began in May 2018 and was estimated to take until mid-2019.

### Trial Status Upon Submission of Protocol Revision

**Figure 2:**
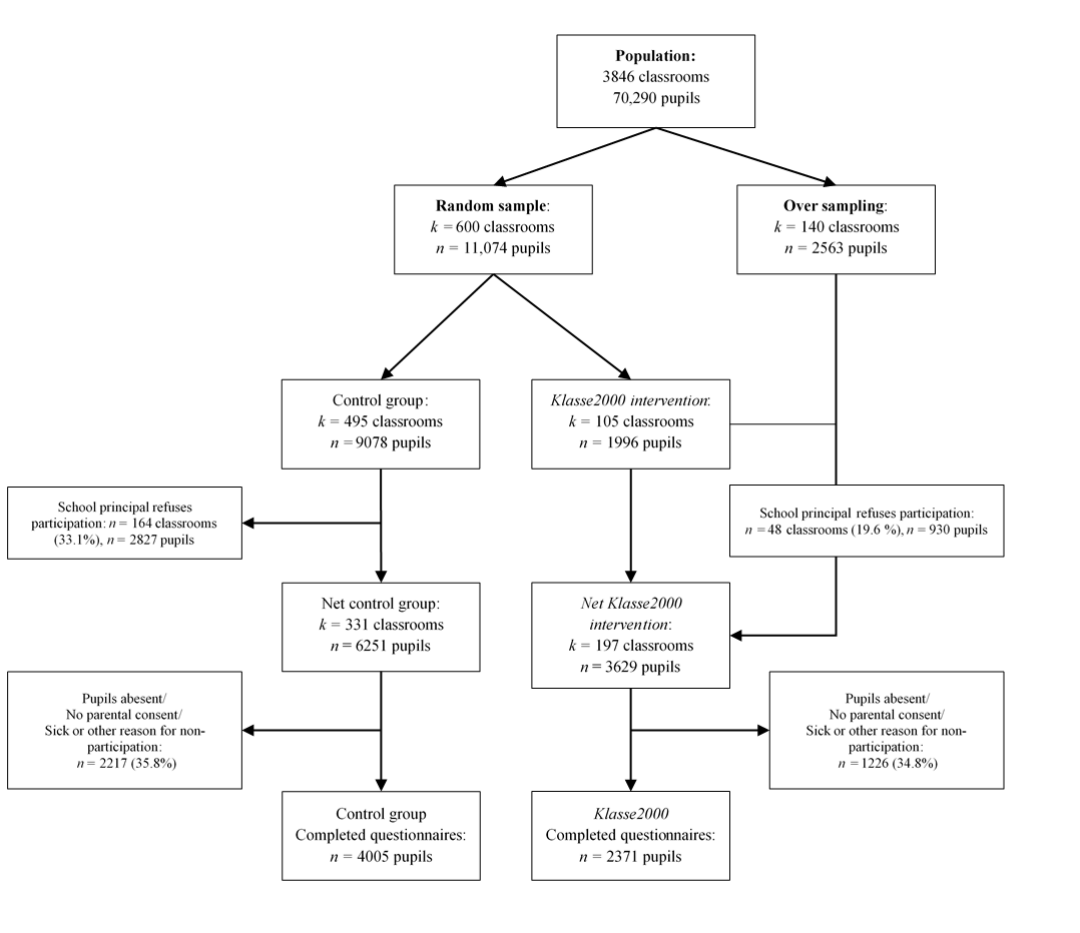
Sampling procedure, participation, and nonparticipation in the Klasse2000 evaluation study.

During the period this protocol was under revision, data integration was completed. Data integration included linking the child and parent data. Matching was performed with the help of the codes. Figure 2 presents the final number of participants and details on nonparticipation. All 6376 participating children were supplied with a questionnaire for their parents. We received 3324 completed questionnaires which corresponds to a response rate of 52.13%. Of the control group, 52.91% (2119/4005) of parents participated in the survey, whereas 50.82% (1205/2371) of parents of the Klasse2000 group returned the questionnaire. Data analysis is ongoing with only descriptive statistics extracted so far. Beyond the descriptives regarding participation, we analyzed the feedback from the test administrators to confirm data quality. Furthermore, the following demographics were obtained: 50.57% (3140/6352) of the children were female. The average age was 10 (SD 0.62) years. The parent survey was mostly completed by a female parent (2899/3259, 88.95%). The parents completing the survey were on average 42.6 years old. Hypothesis-related results are neither obtained nor published upon resubmission of the protocol to ensure transparent reporting and analyses practices.

## Discussion

### Summary

The aim of the study is a large-scale evaluation of the effectiveness of the Klasse2000 prevention program. The effects of the program as implemented in the field shall be evaluated using a representative sample. As a randomized design was not feasible within the framework of this evaluation, a propensity score-matching approach is used to control for potential selection bias.

Intervention effects in the following domains are assessed: well-being, health-related behavior, classroom atmosphere, school violence, alcohol and tobacco consumption, and media use. These domains are investigated from the perspective of the students and their parents. Klasse2000 is a large universal prevention program. It is financially supported with donations from well-known institutions such as health care providers and banks and endorsed by experts of national recognition [21]. The program has received the highest possible rating category from the green list prevention provided by the Crime Prevention Council of Lower Saxony. Furthermore, it has received several awards [21]. Given this high status, the results of this study potentially have far-reaching consequences not only for the program at hand but, depending on the nature of results, on other preventions with similar target areas. It is likely that this evaluation will not confirm all hypotheses stated in this protocol. The failure to find and intended intervention effect might be due to a lack of effect or the presence of a meaningful but undetectable effect. Should the intervention fail to produce some of the desired effects, it might warrant improvement. The materials of the Klasse2000 intervention are regularly updated. These updates attempt to improve support for teachers in the implementation, communication with parents, and communication of teaching content to the students. Recent updates include, for example, content for DigiBoards (Digi International) and an interactive website for the students; further digitalization and extension of online health-related content is planned [21]. Depending on the areas that might warrant improvement, successful concepts from eHealth interventions might provide fruitful starting points. Through the wide implementation of the Klasse2000 program, even small effects might be of value from a public health perspective. Future evaluation studies might want to address this issue using assessment tools that assess the targeted health-related behaviors more reliably. Expertise with mobile assessment from the eHealth domain could help future evaluations to quantify small effects.

As outlined in the introduction, this evaluation is part of an ongoing evaluation effort of the Klasse2000 intervention. With the timepoint of evaluation in the 4th grade, this evaluation addresses short-term effects. To tentatively quantify medium-term effects, results from a student survey among 9th grade students will be used. The study among 9th grade students did not specifically focus on the target areas but assessed similar domains relating to criminal and health-related behavior. Students from the 9th grade survey indicated whether they had participated in the Klasse2000 prevention program when they attended primary school.

### Limitations

The main limitation of the study is the missing randomization. The main problems of missing randomization are potential selection effects at the school level. Schools that are problem-prone might be more likely to participate in the Klasse2000 program, hoping that participation might reduce prevailing problems. On the other hand, the exact opposite might also be the case. Schools that can be characterized as especially committed and dedicated might decide to participate in the Klasse2000 program. Such selection effects might be counterbalanced with the propensity score-matching approach. It should also be noted that the study design does not include any measurement prior to the intervention. This is a cross-sectional survey that can identify correlations but cannot map causal relationships. The results should be checked within the framework of a longitudinal study design and a preliminary survey and offer necessary space for future research projects.

## Appendix

Multimedia Appendix 1 Chain of effects of Klasse2000.

Multimedia Appendix 2 Schedule of enrollment and assessments.

Multimedia Appendix 3 Standard Protocol Items: Recommendations for Interventional Trials (SPIRIT) checklist.

Multimedia Appendix 4 Ethics approval.

Multimedia Appendix 5 Approval of the state school authority.

Multimedia Appendix 6 Letter to the school principal.

Multimedia Appendix 7 Assessment tools.

Multimedia Appendix 8 Parent information letter.

Multimedia Appendix 9 Grant letter.

## Abbreviations

BZgA: Bundeszentrale für Gesundheitliche Aufklärung (Federal Center for Health Education)
IFT-Nord: Institut für Therapie- und Gesundheitsforschung (Institute for Therapy and Health Research)
KFN: Kriminologisches Forschungsinstitut Niedersachsen (Criminological Research Institute of Lower Saxony)
KiGGS: Studie zur Gesundheit von Kindern und Jugendlichen in Deutschland (Study of the Health of Children and Adolescents in Germany)
SDQ: Strength and Difficulties Questionnaire (German version)
SPIRIT: Standard Protocol Items: Recommendations for Interventional Trials
