# Supplementary material for: Evaluation of the Universal Prevention Program Klasse2000 in Fourth Grade Primary School Children: Protocol for a Propensity Score-Matching Approach
Source: JMIR Res Protoc. 2020 Aug 20;9(8):e14371. doi: 10.2196/14371 (PMC7471893; doi:10.2196/14371)
Supplement: Multimedia Appendix 1 [file resprot_v9i8e14371_app1.docx]

Children

The children's health and life skills are strengthened:

- Children know their body and what they can do to stay healthy and feel good, e.g. nutrition, exercise, relaxation
- Children regard health as important and know how they can contribute to their own health
- They acquire important life skills, e.g. dealing with emotions and stress, communicating and cooperating with others, saying no, e.g. to tobacco and alcohol

Acquisition and training of suitable health promoters, administration

 Health promoters

The family supports and reinforces the Klasse2000 domains at home

Health promoters motivate the children; their visits remind teachers of the implementation of the program.

 Parents

**Parents' work:** parents' evenings, letters, newsletters, take home tasks

- “Schule 2020" flyer on school development
- Klasse2000 certification for intensive program implementation

 Schools

Health promotion not only takes place in the classroom, but throughout the school

**Resources (Input)**

- The program Klasse2000 e. V.: Teaching concept, administration, material production and distribution, evaluation, public relations
- Donations and funders enable the program implementation
- Partners and supporters at the regional, state and national level draw schools' attention to the programme and help in obtaining funding.

**Vision and Goals**

*Klasse2000* aims to help children develop a positive attitude towards themselves as well as acquire basic knowledge and coping-skills. The program hopes to exert a positive influence on the children’s social, mental and physical wellbeing.

**Programme implementation by trained Klasse2000 health promoters and teachers**

**Domains:**

- Healthy food and beverage choices
- Exercise and relaxation
- Positive self-image and friendships
- Solving problems and conflicts

Critical thinking and saying no (especially regarding alcohol and tobacco)

**The societal challenge**

The reduction of health risks, prevention and the promotion of a healthy lifestyle.

**Output**

**Target groups**

**Effects**
