# Supplementary material for: Evaluation of the Universal Prevention Program Klasse2000 in Fourth Grade Primary School Children: Protocol for a Propensity Score-Matching Approach
Source: JMIR Res Protoc. 2020 Aug 20;9(8):e14371. doi: 10.2196/14371 (PMC7471893; doi:10.2196/14371)
Supplement: Multimedia Appendix 2 [file resprot_v9i8e14371_app2.docx]

Multimedia Appendix 2: Schedule of enrollment and assessments.

| **TIMEPOINT** | ***12/***  ***2016*** | ***03/***  ***2017*** | ***04/***  ***2017*** | ***06/***  ***2017*** | ***11/***  ***2017*** | ***01/***  ***2018*** | ***12/***  ***2018*** | ***06/***  ***2019*** |
| --- | --- | --- | --- | --- | --- | --- | --- | --- |
| **ENROLMENT:** |  |  |  |  |  |  |  |  |
| **Sampling** | X |  |  |  |  |  |  |  |
| **Test administrator training** |  | X |  |  |  |  |  |  |
| **Letter to principal and scheduling of test administration** |  | X |  |  |  |  |  |  |
| **ASSESSMENTS AND DATA INTEGRATION:**  **Student Survey**  - wellbeing  - self-worth  - emotion regulation  - eating habits  - behavioral strengths and problems  - atmosphere in school and classroom  - bullying and victimization  - education  - school anxiety  - school absenteeism  - alcohol consumption  - smoking  - delinquency  - parental care  - leisure activities  - media equipment  - media use  **Parent survey**  - socio demographics  - wellbeing  - self-esteem  - eating habits  - behavioral strengths and problems  - parenting style  - school absence due to sickness  - media equipment  - media use  - knowledge of *Klasse2000*  - *Klasse2000* components  - participation in *Klasse2000* |  |  |  |  |  |  |  |  |
| ***DATA ANALYSIS*** |  |  |  |  |  |  |  |  |
| ***DISSEMINATION OF RESULTS*** |  |  |  |  |  |  |  | X |
