# Supplementary material for: Evaluation of the Universal Prevention Program Klasse2000 in Fourth Grade Primary School Children: Protocol for a Propensity Score-Matching Approach
Source: JMIR Res Protoc. 2020 Aug 20;9(8):e14371. doi: 10.2196/14371 (PMC7471893; doi:10.2196/14371)
Supplement: Multimedia Appendix 5 [file resprot_v9i8e14371_app5.docx]

Multimedia Appendix 5: Approval of the state school authority.

State school authority of Lower Saxony • Regional department Hanover

Post office box 37 21 • 30037 Hanover

Criminological Research Institute of Lower Saxony

For the attention of: Mr. PD Dr. Mößle

Lützerodestraße 9

30161 Hanover

Processed by

Roman Lemke

Regional department Hanover

[Roman.Lemke@nlschb.niedersachsen.de](mailto:Roman.Lemke@nlschb.niedersachsen.de)

Fax: 0511 106-2443

Your sign, your message from My sign (indicate on answer) Telephone number

13 February 2017; 27 February 2017 **H 1 R b – 81402 – 09 – 2017** 0511 106-2443

Hanover

27 February 2017

**Polls and school surveys**

**RdErl. D. MK. B. 1.12.2015 – 26 – 81402 – Voris 22410**

**Approval of the Lower Saxony-wide student survey 2017 in fourth classes for the evaluation of the prevention programme Klasse2000**

Dear Mr. PD Dr. Mößle,

I hereby authorize your request to conduct the above-mentioned survey in those schools that you have named to me in a school list. The permit only applies to public schools. In the case of independent schools, the respective school authorities decide on the conduct of the survey. An integration of further schools is not included in this permit.

Concerning the details of the project and its implementation, I refer to the information you have provided in your application.

The regulations in the decree of the Ministry of Culture of Lower Saxony (MK) “Polls and surveys in schools” of the 1^st^ December 2015 (SVBI. No.12/2015), p. 598) already known to you, apply to the implantation of the project, which I ask you to follow.

The approval is given with the note that participation in the survey is voluntary. Voluntariness also includes the right to answer single questions and omit others. Teachers must be informed of this in advance.

I also ask that the anonymity of those involved in the investigation is protected and that personal data - insofar as they are collected - as well as the results are protected under data protection law. The survey must not interfere with personal rights worthy of protection; for example, the survey must not lead to discrimination against individual persons.

Personal data is to be collected anonymously. If this is not possible from the outset, the data must be made anonymous as soon as this is possible without impairing the success of your examination.

In case of any publications about this project, I ask you to ensure that it is not possible to draw conclusions about the respective school and the school management.

I also assume that the survey will have as little impact as possible on lessons. The organisational measures required to carry out the survey at the school are to be agreed with the school management and require their approval.

I would also like to point out that according to No. 7 of the decree, schools decide on their participation in an approved survey under their own authority.

I note that there is no financial obligation for the State of Lower Saxony as a result of this approval.

I wish you every success in the implementation of your project.

I ask you to inform me about the results of the survey in due course and to send a copy of the results to the Ministry of Culture of Lower Saxony, Schiffgraben 12, 30159 Hanover.

With kind regards

On behalf

Roman Lemke
