# Supplementary material for: Evaluation of the Universal Prevention Program Klasse2000 in Fourth Grade Primary School Children: Protocol for a Propensity Score-Matching Approach
Source: JMIR Res Protoc. 2020 Aug 20;9(8):e14371. doi: 10.2196/14371 (PMC7471893; doi:10.2196/14371)
Supplement: Multimedia Appendix 6 [file resprot_v9i8e14371_app6.docx]

Multimedia Appendix 6: Letter to the principal.

| CRIMINOLOGICAL 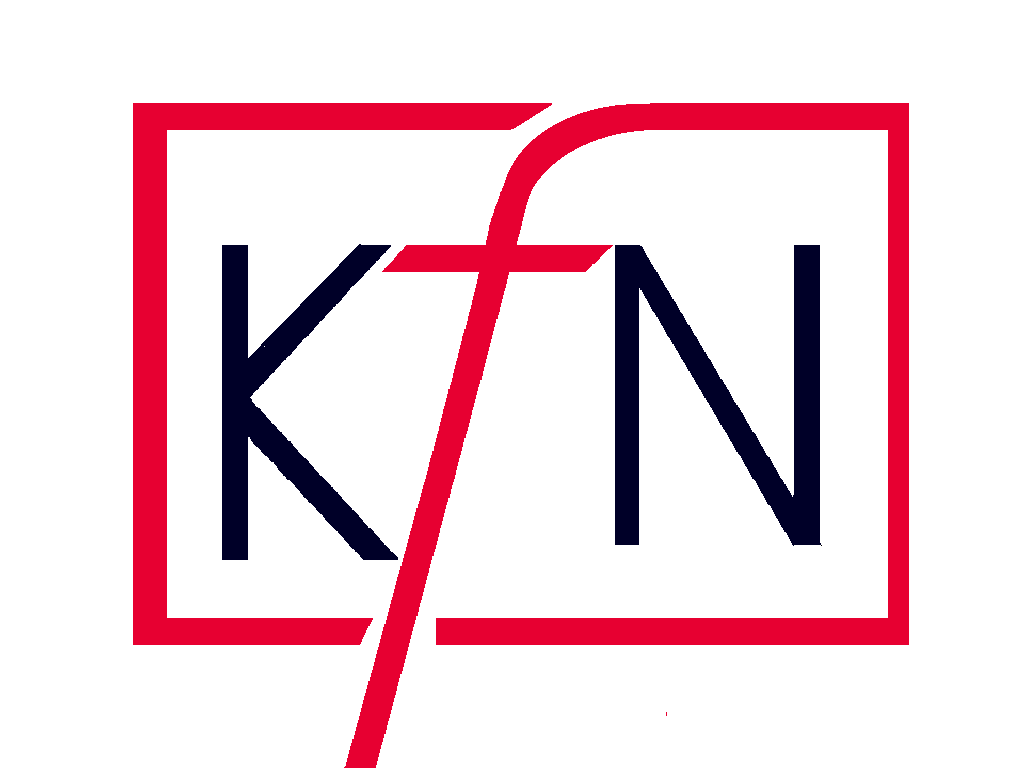 RESEARCH INSTITUTE OF  LOWER SAXONY | |
| --- | --- |
| **Criminological Research Institute of Lower Saxony, Lützerodestraße 9, 30161 Hannover**  To the principal’s office  Adress of the school |  |

March 15^th^ 2017

**Announcement of the student survey of the fourth grades in Lower Saxony**

Dear principal,

The Criminological Research Institute of Lower Saxony (KFN) is conducting a comprehensive survey of fourth graders funded by the Federal Centre for Health Education (BZgA). The most important topics covered by the planned survey are violence prevention, general life skills and the use of on-screen media. With a planned survey of around 600 fourth grades in Lower Saxony, we expect to reach around 8,000 pupils. The KFN has extensive expertise in the area of student surveys (e.g., the Lower Saxony survey with 13,000 pupils).

The forthcoming survey is to take place between **24 April 2017 and 21 June 2017**. For this purpose, the class(es) 4x was/were randomly selected from your school. Participation in this survey is voluntary for the students. The data obtained are treated with absolute confidentiality and anonymity is guaranteed at all times.

The purpose of this letter is to inform you about the planned student survey so that you can integrate it better into the lesson plan. We try our best to keep the organizational effort for you and the teachers of the above-mentioned classrooms as low as possible. The following steps would be necessary for the implementation of the survey:

- The teacher(s) and parents would be informed about the survey via a letter. A sufficient number of these letters would be made available to your school by the KFN.
- Test administrators, who are employed and trained especially for the survey, would arrange a possible interview date with the teacher(s). The survey will take place during school hours and will last about one hour. In preparation for the interview, the responsible test administrator would contact you in due time and answer all remaining questions.
- The students will furthermore be provided with a questionnaire for their parents. There will also be a questionnaire for you as principal, which we would ask you to complete.

If you have any questions regarding the planned study, please contact us at 0511-34836-70 or via email to Yvonne.Krieg@kfn.de. We would be very grateful for your participation and support.

Sincerely,


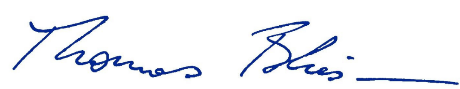

Prof. Dr. Thomas Bliesener (Director) Dr. Sören Kliem (Project lead)
