# Supplementary material for: Evaluation of the Universal Prevention Program Klasse2000 in Fourth Grade Primary School Children: Protocol for a Propensity Score-Matching Approach
Source: JMIR Res Protoc. 2020 Aug 20;9(8):e14371. doi: 10.2196/14371 (PMC7471893; doi:10.2196/14371)
Supplement: Multimedia Appendix 7 [file resprot_v9i8e14371_app7.docx]

**Multimedia Appendix 7. Assessment Tools**

**Table 1.** List of assessment tools of the *Klasse2000* evaluation.

| Hypothesis | Construct | Operationalization | Primary or secondary outcome domains | Informant |
| --- | --- | --- | --- | --- |
| Hypothesis 1.1: The prevention program Klasse2000 has a positive influence on the children’s wellbeing. | Wellbeing | Inventory for the assessment of quality of life in children and adolescents (ILK) [Inventar zur Erfassung der Lebensqualität bei Kindern und Jugendlichen] | Primary outcome domain | Students  Parents |
| Hypothesis 1.2: The prevention program Klasse2000 has a positive influence on the children’s self-esteem. | Self-esteem | Self-esteem dimension of the questionnaire for the assessment of health-related quality of life in children (KINDL) [Fragebogen zur Erfassung der gesundheitsbezogenen Lebensqualität von Kindern und Jugendlichen] | Primary outcome domain | Students  Parents |
| Hypothesis 1.3: The prevention program Klasse2000 has a positive influence on the children’s emotion regulation. | Emotion regulation | Instrument to measure emotion regulation strategies in children and adolescents [Fragebogen zur Erhebung der Emotionsregulation bei Kindern und Jugendlichen] (FEEL-KJ) | Primary outcome domain | Students |
| Hypothesis 1.4: The prevention program Klasse2000 reduces the severity of behavioral problems. | Behavioral problems | Strengths and Difficulties Questionnaire German translation (SDQ-Deu) | Primary outcome domain | Students  Parents |

| Hypothesis | Construct | Operationalization | Primary or secondary outcome domains | Informant |
| --- | --- | --- | --- | --- |
| Hypothesis 2.1: The prevention program Klasse2000 increases fruit and vegetable consumption. | Eating habits | Klasse2000 evaluation by the IFT-Nord | Primary outcome domain | Students  Parents |
| Hypothesis 2.2: The prevention program Klasse2000 increases the consumption of water and unsweetened tea. | Eating habits | Klasse2000 evaluation by the IFT-Nord | Primary outcome domain | Students  Parents |
| Hypothesis 2.3 The prevention program Klasse2000 decreases the consumption of sweets, salty snacks (i.e., chips, pretzels…) and sweetened beverages. | Eating habits | Klasse2000 evaluation by the IFT-Nord | Primary outcome domain | Students  Parents |
| Hypothesis 2.4 The prevention program Klasse2000 increases the time children spend exercising. | Exercise | KFN student survey | Primary outcome domain | Students  Parents |
| Hypothesis 3.1: The prevention program Klasse2000 has a positive influence on the school and classroom atmosphere. | Classroom atmosphere | Linzer School-and classroom atmosphere Questionnaire (LFSK 4-8) [Linzer Fragebogen zum Schul- und Klassenklima] | Primary outcome domain | Students |
| Hypothesis | Construct | Operationalization | Primary or secondary outcome domains | Informant |
| Hypothesis 3.2: The prevention program Klasse2000 decreases the probability of engaging in bullying. | School violence and bullying | Bullying and victimization self-report for children (BVF-K) [Bullying- und Viktimisierungsfragebogen für Kinder] | Primary outcome domain | Students |
| Hypothesis 3.3: The prevention program Klasse2000 decreases the probability of becoming a victim of bullying. | School violence and bullying | Bullying and victimization self-report for children (BVF-K) [Bullying- und Viktimisierungsfragebogen für Kinder] | Primary outcome domain | Students |
| Hypothesis 4.1: The prevention program Klasse2000 decreases the time spent on media. | Media consumption | KFN student survey | Secondary outcome domain | Students  Parents |
| Hypothesis 4.2: The prevention program Klasse2000 decreases the probability of watching movies with an age rating of 16 or 18. | Media content | Berlin Longitudinal Study Media | Secondary outcome domain | Students |
| Hypothesis 4.3: The prevention program Klasse2000 decreases the frequency of watching movies with an age rating of 16 or 18. | Media content | Berlin Longitudinal Study Media | Secondary outcome domain | Students |

| Hypothesis | Construct | Operationalization | Primary or secondary outcome domains | Informant |
| --- | --- | --- | --- | --- |
| Hypothesis 4.4: The prevention program Klasse2000 decreases the probability of playing video games with an age rating of 16 or 18. | Media content | Berlin Longitudinal Study Media | Secondary outcome domain | Students |
| Hypothesis 4.4: The prevention program Klasse2000 decreases the frequency of playing video games with an age rating of 16 or 18. | Media content | Berlin Longitudinal Study Media | Secondary outcome domain | Students |
| Hypothesis 5.1: The prevention program Klasse2000 decreases the probability of drinking alcohol. | Alcohol consumption | Berlin Longitudinal Study Media | Secondary outcome domain | Students |
| Hypothesis 5.2: The prevention program Klasse2000 decreases the frequency of alcohol consumption. | Alcohol consumption | Berlin Longitudinal Study Media | Secondary outcome domain | Students |
| Hypothesis 5.3: The prevention program Klasse2000 decreases the probability of smoking cigarettes. | Tobacco consumption | Berlin Longitudinal Study Media | Secondary outcome domain | Students |

| Hypothesis | Construct | Operationalization | Primary or secondary outcome domains | Informant |
| --- | --- | --- | --- | --- |
| Hypothesis 5.4: The prevention program Klasse2000 decreases the smoking frequency. | Tobacco consumption | Berlin Longitudinal Study Media | Secondary outcome domain | Students |

### Deployed Measuring Instruments

Children were presented with an 11-page questionnaire. The teacher as well as the test administrator were present during the entire assessment. The test administrator led the children through the questionnaire following a written manual. All questions were projected in the classroom. The test administrator read each question as well as the possible answers aloud and provided additional explanation for more complex items. Figure 2 provides an example of the questionnaire layout. The following section provides an overview of all instruments that were part of the assessment.


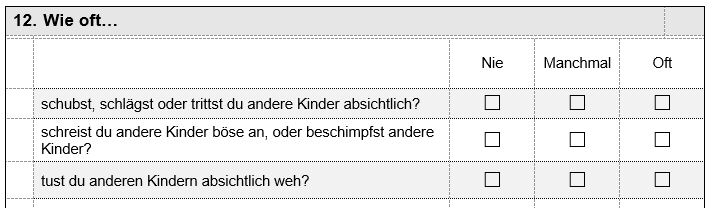


Fig. 1 Partial question from the student questionnaire for layout illustration

**Research question 1: Mental Health**

***Quality of life.*** For the assessment of the children’s wellbeing the Inventory for the assessment of quality of life in children and adolescents (ILK) [Inventar zur Erfassung der Lebensqualität bei Kindern und Jugendlichen] was used [1]. The ILK comprises seven items assessing the domains school, family, social contact to peers, hobbies and leisure activities as well as physical and mental health in addition to one item for general well-being. The inventory is administered in a student as well as in a parent version. Parents assess the seven domains on a five-point scale ranging from “1-very good“ to “5-very bad“. The children also answer the items on a five-point scale that is represented by happy or sad smiley faces. Furthermore, the parents assessed the priority of each domain for their child. Example item: „If you are on your own, for example when you play by yourself or do something else, how does that make you feel?”

***Self-esteem.*** The children’s self-worth is assessed with the self-worth subscale of the Kindl questionnaire for the assessment of health-related quality of life in children [Fragebogen zur Erfassung der gesundheitsbezogenen Lebensqualität von Kindern und Jugendlichen] [2,3]. The questionnaire has proven to be highly accepted with children and adolescents [2]. In a study among 1050 children suffering from chronic diseases and their parents [3] the self-worth subscale showed a scale fit (the percentage of items that correlate higher with their intended subscale instead of a different one) of 100% and Cronbach’s alpha of 0.75 (children) and. 0.77 (parents). The self-worth dimension consists of four items that are answered on 5-point scale ranging from “1-never“ to “5-always“. Parents as well as children answer items with respect to the week prior to the survey. Example item: “During the last week I was proud of myself”

***Emotion regulation.*** Emotion regulation is assessed with the instrument to measure emotion regulation strategies in children and adolescents FEEL-KJ [Fragebogen zur Erhebung der Emotionsregulation bei Kindern und Jugendlichen] [4]. It assesses three *adaptive coping strategies* (problem-oriented behavior, mood improvement, cognitive problem solving) as well as three *maladaptive coping strategies* (resignation, aggressive behavior, self-degradation). In addition to these six subscales social-support is assessed with two further items which cannot be allocated to the two secondary scales. Altogether, the children answer a total of 14 items that describe behavior in times of being angry, sad or scared on a five-point scale ranging from “1-almost never” to “5 almost always”. The FEEL-KJ is evaluated based on the six strategy subscales as well as the two secondary dimensions *adaptive and maladaptive coping*. Reliability as indicated by Cronbach’s alpha can be regarded as satisfactory. For the *adaptive strategies* Cronbach’s alpha is reported as α = .73 (problem-oriented behavior) and α = .88 (mood improvement). The overall score of the *adaptive strategies* scale showed an internal consistency of α = .93 For the *maladaptive strategies* subscale reliabilities between α = .69 (resignation) and α = .78 (aggressive behavior) are reported. The sum-scale for *maladaptive strategies* showed an alpha of α = .82. Example item: “When I am angry, sad or frightened I tell someone how I feel”

***Problem behavior.*** Problem behavior in children is assessed using the German translation of the strengths and difficulties Questionnaire (SDQ-Deu) [5,6]. The SDQ is a screening tool for the assessment of behavioral strengths and problems in children and adolescents between the ages of 4 and 16 [5]. It consists of five subscales that cover the dimensions: *emotional symptoms, conduct problems, hyperactivity-inattention, peer problems und prosocial behavior.* The questionnaire is administered in a self-report version as well as in a parent-version. The 24 items that cover the child’s behavior in the last six months are rated on a 3-point scale ranging from “0- not true“ to “2- certainly true“. The total difficulties score is obtained by summing all subscales except for the *prosocial behavior* scale and can range from 0 to 40 [6]. Previous studies have shown more reliable results using the total difficulties score. To further assess the validity of the instrument the results were compared to the Child Behavior Checklist (CBCL) [7,8] which is five times as long. Both instruments show high correlations of child and parent version indicating a high convergent validity [6].

**Research question 2: Health related behavior**

***Eating habits.*** In accordance with the *Klasse2000* evaluation carried out by the IFT-Nord [9], children and parents are asked to provide information regarding the eating habits of their child. On a four point scale they indicate the amount of fruit and vegetables (“1 - none” to ”4 - five or more portions“) as well as water and unsweetened tea (“1 - none” to “4 - six or more glasses”). Furthermore the amount of sweats and salty snacks is assessed (“1-none to “4-three or more portions“).

***Exercise.*** The exercise behavior was assessed in accordance with the KFN student surveys [10]. Exercise is assessed as the number of hours that the children spent engaging in sport outside of school (as indicated by both children as well as parents). The children indicated their exercise behaviors on a seven point scale ranging from “1- I do not do this at all“ to “7-more than three hours”. The parents could furthermore indicate the number of hours their child usually spends on a certain activity. Both, parents as well as children, were asked to indicate the frequency on schooldays and on a weekend day separately. In accordance with Bergmann et al. (2017) the number for the school day is multiplied by five and for the weekend by two and then divided by seven in order to calculate the average length of the activity per day [10].

**Research question 3: School and classroom atmosphere, conflicts and bullying**

***Classroom atmosphere.*** The classroom atmosphere was assessed with an adapted version of the Linzer school-and classroom atmosphere questionnaire (LFSK 4-8) [Linzer Fragebogen zum Schul- und Klassenklima] [11]. The present study uses the two subscales *Community* and *Rivalry* with three items each. Students rate the items on a 5-point scale ranging from “1-not true“ to “5-entirely true“. Example item: “In our school students are happy to help each other.”

***Conflicts und Bullying.*** For the assessment of victimization as well as perpetration with regard to bullying the bullying and victimization self-report for children (BVF-K) [Bullying- und Viktimisierungsfragebogen für Kinder im Selbstbericht] [12] is used. The BVF-K is an assessment tool for use in kindergarten and primary school. It assessed *direct and indirect victimization* as well as *indirect and direct aggression* with eight items each. Children rate their answers on a three point scale ranging from “1-never“ to “3-often“. In a sample of second grade students the internal reliability for the victim and perpetrator scale was reported as Cronbach’s α =.77 and α =.90. The subscales showed internal consistencies of α = .60 to α = .77 [12]. Example item: ”How often does another child force you to do something that you don’t want to do?”

**Research question 4: Media usage**

***Media usage.*** Time spent with electronic media is assessed in accordance with the Berlin Longitudinal Study Media [Längsschnitt Medien] [13]. Children as well as their parents indicate the number of hours that are usually spent on: *watching TV and movies (offline)*, *playing video games*, *spending time on social media and chatting*. The self-report version for the children ranged from “1- I do not do that at all“ to “7-more than three hours”. The parents could indicate the number of hours and minutes in a text box. They furthermore had the option to indicate that their child did not engage in the activity at all. As with the assessment of exercise behavior, the frequency was assessed separately for a typical school day and for a typical day on the weekend. In accordance with Bergmann et al. (2017) the number for schooldays was multiplied by five and the number for a typical weekend day by two and then divided by seven in order to calculate the average media usage time per day [7].

***Media content.*** As in the Berlin Longitudinal Study Media [13], students are asked whether they had ever watched a movie with an age rating of 16 or 18 or played a video game with an age rating of 16 or 18. If this question is affirmed the students are asked about the frequency of this behavior in the previous four weeks.

**Research question 5: Alcohol and tobacco consumption**

***Alcohol and tobacco consumption.*** Alcohol and tobacco consumption is assessed via self-report as in the Berlin Longitudinal Study Media [13]. Children are asked if they had ever consumed more than a sip of alcohol or smoked more than one cigarette. If any one of these behaviors is affirmed the frequency of said behavior in the previous four weeks is assessed.

### References

1. Mattejat F, Remschmidt H. Das Inventar zur Erfassung der Lebensqualität bei Kindern und Jugendlichen (ILK): [The inventory of life quality in children and adolescents (ILC)]. Bern: Verlag Hans Huber; 2006.

2. Ravens-Sieberer U, Bullinger M. Assessing health-related quality of life in chronically ill children with the German KINDL: first psychometric and content analytical results. Quality of Life Research 1998;7(5):399-407. PMID: 9691720

3. Ravens-Sieberer U, Bullinger M. Kindl. Fragebogen zur Erfassung der gesundheitsbezogenen Lebensqualität bei Kindern und Jugendlichen.: Revidierte Form. Manual; 2000.

4. Grob A, Smolenski C. FEEL-KJ: Fragebogen zur Erhebung der Emotionsregulation bei Kindern und Jugendlichen. 2., aktualisierte und ergänzte Auflage. Bern: Hans Huber; 2009.

5. Goodman R. The Strengths and Difficulties Questionnaire: A Research Note. J Child Psychol & Psychiat 1997;38(5):581-586. PMID: 9255702

6. Klasen H, Woerner W, Wolke D, Meyer R, Overmeyer S, Kaschnitz W, Rothenberger A, Goodman R. Comparing the German Versions of the Strengths and Difficulties Questionnaire (SDQ-Deu) and the Child Behavior Checklist. European Child & Adolescent Psychiatry 2000;9(4):271-276. PMID: 11202102

7. Achenbach TM. Manual for the Child Behavior Checklist/4-18 and 1991 Profile. Burlington: University of Vermont, Department of Psychiatry; 1991.

8. Arbeitsgruppe Deutsche Child Behavior Checklist. Elternfragebogen über das Verhalten von Kindern und Jugendlichen: Deutsche Bearbeitung der Child Behavior Checklist (CBCL/ 4-18).: Einführung und Anleitung zur Handauswertung. 2. Auflage mit deutschen Normen. Köln: Arbeitsgruppe Kinder-, Jugend-, und Familiendiagnostik; 1998.

9. Maruska K, Isensee B, Hanewinkel R. Das Unterrichtsprogramm Klasse2000: Welche Effekte gibt es über die Grundschulzeit hinaus? Kiel: Institut für Therapie- und Gesundheitsforschung gGmbH; 2010.

10. Bergmann MC, Baier D, Rehbein F, Mößle T. Jugendliche in Niedersachsen: Ergebnisse

des Niedersachsensurveys 2013 und 2015. Hannover: Kriminologisches Forschungsinstitut Niedersachsen; 2017.

11. Eder F, Mayr J. Linzer Fragebogen zum Schul-und Klassenklima für die 4.-8. Klasse:(LFSK 4-8): Hogrefe; 2000.

12. Petermann F, Marée N von. Der Bullying- und Viktimisierungsfragebogen für Kinder (BVF-K): Konstruktion und Analyse eines Verfahrens zur Erhebung von Bullying im Vor- und Grundschulalter. [The bullying and victimization questionnaire for children (BVF-K): construction and analysis of an instrument for the assessment of bullying in kindergarten and primary school]. Praxis der Kinderpsychologie und Kinderpsychiatrie 2009;58(2):96-109. PMID: 19334400

13. Mößle T. Dick, dumm, abhängig, gewalttätig?: Problematische Mediennutzungsmuster und ihre Folgen im Kindesalter; Ergebnisse des Berliner Längsschnitt Medien. 1. Aufl. Baden-Baden: Nomos; 2012. ISBN:978-3-8329-7499-2.
