# Supplementary material for: Evaluation of the Universal Prevention Program Klasse2000 in Fourth Grade Primary School Children: Protocol for a Propensity Score-Matching Approach
Source: JMIR Res Protoc. 2020 Aug 20;9(8):e14371. doi: 10.2196/14371 (PMC7471893; doi:10.2196/14371)
Supplement: Multimedia Appendix 8 [file resprot_v9i8e14371_app8.doc]

Multimedia Appendix 8: Parent information letter.


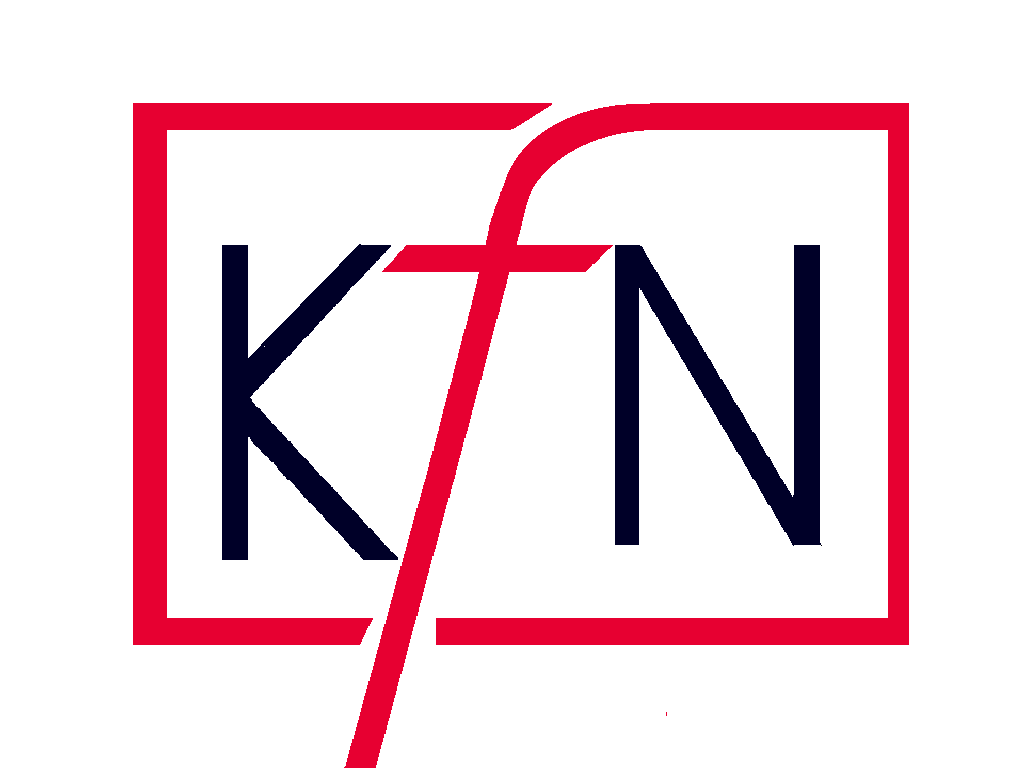


CRIMINOLOGICAL

Lützerodestraße 9 · D-30161 Hannover

Tel. 0511-3 48 36-0 · E-Mail: kfn@kfn.de

RESEARCH INSTITUTE

OF LOWER SAXONY

**Parent information: Student survey of the fourth grades in Lower Saxony**

Dear parents,

Problems concerning children and adolescent often dominate the headlines in the press and on television. The increase in juvenile violence or the problematic use of media are current issues. At the same time, we have to concede that little is known about the real extent of these problems, their development and their causes.

The Criminological Research Institute of Lower Saxony (KFN) has therefore decided to conduct a comprehensive survey of fourth grade students to further investigate these issues. The survey is financed by the Federal Centre for Health Education (BZgA). In total, about 8,000 children from all over Lower Saxony will be assessed with regard to various aspects of their daily lives. This includes their attitudes toward school and leisure behavior as well as information about age and gender. Questions are asked regarding the parents’ or respondents' origins, experiences with violence, the parenting style in the family, the well-being of the children, alcohol and cigarette consumption, nutrition and the use of consumer electronics.

The survey will be conducted in the next few days. It will take place during school hours and will last about one hour. Participation is entirely voluntary; there are no disadvantages if your child does not participate. Your child can decide for every single question whether he or she does or does not want to provide an answer. Your child will not be asked for any identifying information such as his or her name and address. The survey will be evaluated anonymously; all information provided by the students will be treated strictly confidential. All individuals who are involved in the survey or its evaluation are sworn to secrecy and data protection. No information about individual students, classes or schools is passed on. After the questionnaires are filled in, they are stored in a sealed envelope on site.

In addition to the questionnaire for the students, there will be a parent questionnaire that your child will bring along from school. This will include questions about your family situation, your child’s leisure activities, consumer electronics and media use as well as the well-being of your child. We kindly ask you to return your completed questionnaire in the prepaid envelope.

We would like to ask you as parents to support this important research project. Please sign the consent form below and ask your daughter/son to return it to their teacher. Students who do not participate in the survey will be properly supervised while their fellow students complete the questionnaires.

Thank you for your support.

Sincerely,


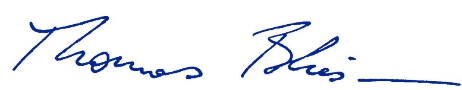

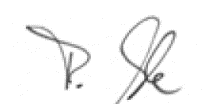


Prof. Dr. Thomas Bliesener Prof. Dr. Thomas Mößle Dr. Sören Kliem

(Director) (Project lead) (Project lead)

**✂----------------------------------------------------------------------------------------------------------------------**

**Statement on participation in the survey**

I allow my daughter/my son ______________________ to participate in the above-mentioned survey during school hours.

_____________________, _____________,__________________________________________

(Place) (Date) (Signature of a Parent or legal guardian)

*The completed consent form will remain with the teacher and is destroyed two weeks after the survey.*
