# Supplementary material for: Evaluation of the Universal Prevention Program Klasse2000 in Fourth Grade Primary School Children: Protocol for a Propensity Score-Matching Approach
Source: JMIR Res Protoc. 2020 Aug 20;9(8):e14371. doi: 10.2196/14371 (PMC7471893; doi:10.2196/14371)
Supplement: Multimedia Appendix 9 [file resprot_v9i8e14371_app9.docx]

Multimedia Appendix 9: Letter of grant from the BZgA.

BZgA • 50819 Köln

Criminological Research Institute of Lower Saxony

Lützerodestr. 9

30161 Hannover

Maarweg 149-161

50825 Köln

Telephone (0221) 8992-0

Fax (0221) 8992-300

Your sign, your message from My sign (indicate on answer) Telephone number Date

15 Februrary 2016 Z2/21.34.20/15 (0221) 8992-215 9 June 2016

**Letter of grant**

**Grants from federal funds in accordance with § 44 BHO**

**Evaluation of the prevention program *Klasse2000* in Lower Saxony**

**Your application from 15 February 2016**

Dear Sir or Madam,

hereby a non-repayable grant up to the amount of 238,110 Euro (in words: two hundred and thirty-eight thousand one hundred and ten euros) is granted as shortfall financing in accordance with § 44 of the Federal Budget Code (BHO) in conjunction with §§ 48, 49 of the Administrative Procedure Act (VwVfG) for the time from 01 April 2016 to 31 December 2019 for the implementation of the above mentioned project.

[…]

With kind regards

On behalf of

Schöller
