# Supplementary material for: Evaluation of the Universal Prevention Program Klasse2000 in Fourth Grade Primary School Children: Protocol for a Propensity Score-Matching Approach
Source: JMIR Res Protoc. 2020 Aug 20;9(8):e14371. doi: 10.2196/14371 (PMC7471893; doi:10.2196/14371)
Supplement: Multimedia Appendix 4 [file resprot_v9i8e14371_app4.pdf]

Georg-August-Universität Göttingen

Fakultät für Biologie und Psychologie

Georg-Elias-Müller-Institut für Psychologie
Ethikkommission

Prof. Dr. Annekathrin Schacht

Goßlerstr. 14
37073 Göttingen

Tel +49 (0) 551 / 39-20625
Fax +49 (0) 551 / 39-13570

ethikkommission@psych.uni-goettingen.de
https://www.psych.uni-goettingen.de/de/ethics

Dr. Sören Kliem
Criminological Research Institute of Lower Saxony

Göttingen, January 26 2018

Vote on application no. 184

Title: *Evaluation Klasse2000 in Lower Saxony*

Dear Dr. Kliem,

The ethics committee of the Georg-Elias-Müller-Institute for Psychology has carefully examined your application for evaluation of the research project and has ruled by a majority that your research project is ethically acceptable.

We wish you a successful completion of this project and emphasize that the ethical and legal responsibility for the implementation of the study remains with the project leader and the project staff, independent of the positive statement of the commission.

Sincerely,

A. Schacht

Prof. Dr. Annekathrin Schacht
[Chairperson of the committee]
